# Errors in the Hematology Laboratory at St. Paul’s Hospital Millennium Medical College, Addis Ababa, Ethiopia

**DOI:** 10.1186/s13104-018-3551-y

**Published:** 2018-07-03

**Authors:** Hirut Tadesse, Kassu Desta, Samuel Kinde, Fatuma Hassen, Addisu Gize

**Affiliations:** 1 Department of Laboratory Science, St. Paul’s Hospital Millennium Medical College, Addis Ababa, Ethiopia; 2 Department of Medical Laboratory Sciences, School of Allied Health Sciences, College of Health Sciences, Addis Ababa University, Addis Ababa, Ethiopia; 3 Department of Microbiology, St. Paul’s Hospital Millennium Medical College, P.O.Box 1271, Addis Ababa, Ethiopia

**Keywords:** Laboratory errors, Hematological errors, Pre-analytical errors in hematology

## Abstract

**Objective:**

The objective of this study was to determine the magnitude of pre-analytical, analytical and post-analytical laboratory errors in hematology tests.

**Results:**

A total of 2606 hematology requests were studied. Out of the total, 562 (21.6%) pre-analytic, 14 (0.5%) analytical and 168 (6.4%) post-analytical errors were recorded which contribute a total frequency of 75.5, 1.9 and 22.6%, respectively. The name of the physician requesting the test was not provided on 2215 (85%) of request forms and 1827 (70.1%) of the request forms were unaccompanied with proper clinical details of the patient. Essential information required on the request forms was often missed. Close communication between clinicians and laboratory personnel is the key to improve laboratory quality in general.

## Introduction

An error in the hematology laboratory may begin when the patient is ready to give specimen for testing, analysis until results are released to the clinician and makes diagnostic and therapeutic decisions according to their interpretation. This whole process is impossible to perform totally free of error. Any laboratory analysis always works to minimize this uncertainty and estimate their size with acceptable degree [1].

The pre-analytical phase of work flow includes a set of processes that take place in different places at different times. The pre-analytical phases include all processes from the time a laboratory request is made by the physician until the sample is ready for testing [2, 3]. The main processes that should be taken into account in the study of the pre- analytical phase are; properly identification of patients and following the right sample collection, transport, store and test selection [4–6]. Out of the three phases errors occurring in the laboratory, the pre-analytical phase account the major figure (46–68%), followed by post-analytical phase (19–47%) of errors [7]. A minority (13–32%) according to the studies, occurred in the analytical portion [8]. The magnitude of the effect of these errors on patient care is not negligible; it may lead to incorrect diagnostic and therapeutic decisions, misinterpretation of results, and impairment of meaningful comments from the clinical laboratory [9, 10], since information provided by clinical laboratories affects up to 60–70% of clinical decisions [11].

The analytical phase begins when the patient specimen is prepared in the laboratory for testing and it ends when the test result is interpreted and verified by the technologist in the laboratory [12]. Errors in this phase may be originated from the equipment itself or from interfering compound of the analytic sample. Analytical errors are classified into random errors and systematic errors. It is clear that random errors indicate poor precision while systematic errors indicate poor accuracy. A few examples of random errors are pipetting error, transcription error, wrong sample numbering and labeling, and fluctuating readings on the colorimeter. Systematic errors could occur due to wrong procedure, incorrect standards and calibration procedures [13].

In post-analytical phase, results are reported to the physicians for their interpretation and to treat the patient. However, careless reporting of results and wrong transcription at this phase leads to post-analytical phase errors [1]. In the post-analytical step, the most common mistakes are wrong validation; delayed results, not reported or reported to the wrong providers, and incorrect results reported because of post-analytical data entry errors and transcription errors are common activities.

The post-analytical procedures performed within the laboratory include verifying laboratory results, feeding them into the laboratory information system, and communicating them to the clinicians in a number of ways, usually by producing a report and making any necessary oral communications regarding ‘‘alert’’ or panic results [14, 15].

The United States agency for healthcare research and quality estimates that medical errors are the 8th leading cause of death in the United States, which is higher than motor vehicle accidents cancer and AIDS events annually [16, 17]. Even though automation, standardization and technological advances have significantly improved the analytical reliability of laboratory tests [12, 15, 18], laboratory errors still do occur in every process. Therefore, the present study aims to fill this gap and generate information on pre analytical, analytical and post analytical errors as well as analyze their distribution across settings.

## Main text

### Methods

A cross sectional study was conducted from December 2014 to March 2015 at St. Paul’s Hospital Millennium Medical College (SPHMMC), Addis Ababa, Ethiopia. The hospital laboratory serves an average 300 patients daily and many patients are referred from different parts of the country for different services.

A structured check list was used to collect information like patient card number, name of patient, ward/clinic name, age of the patient, clinical detail, physician name and signatures, the presence and absence of hemolysis, clotting, and inadequate samples, etc.

The check list was pre-tested before the actual data collection in the selected hospital to ensure the validity of the study tool. Then, appropriate modifications were made to standardize the tool. The principal investigator was supervised the whole work during collection procedure.

Two data collectors were trained for 2 days about how to use the check list. Storage condition, acceptable and rejection sample criteria were some of the points discussed with data collectors. In brief, they were oriented that samples to be processed for hematology must be run within 24 h, if kept at room temperature and 36 h, if kept at 4 °C. No special preparation of the patient is necessary. For erythrocyte sedimentation rate, specimens must be run within 4 h. Samples were considered as acceptable in hematology for this study; the samples must be collected in correct sample container (LAVENDER TOP K2 EDTA anticoagulant tubes), correct specimen volume, i.e., all EDTA tubes are optimized at 1.5 mg. EDTA/ml of whole blood. The minimum amount suggested by BD, the tube vendor is at least a 90% draw volume. For example, the 6.0 ml EDTA tubes should have at least 5.4 of whole blood and the 3.0 ml EDTA tubes should have at least 2.7 ml of whole blood.

However, reasons to reject samples were also noticed like, clotted specimen, hemolyzed specimen, improperly labeled or unlabeled specimen, leaking tubes and delay in transport.

Data gathered in the study period were cleaned and double entered into computer using Excel sheet. The analysis was done using SPSS version19. Percentage and frequency was calculated.

Ethical approval was obtained from the Department of Medical Laboratory Science, College of Health Science, Addis Ababa University Research and Ethical Review Committee. Informed written permission was obtained from St. Paul’s Hospital Millennium Medical College Institutional Review Board (IRB) and submitted to the head of the laboratory department.

### Results

A total of 2606 hematology request forms were collected from outpatient department (OPD), private wings, and emergency and in patient departments. Of the total tests requested 386 (15%) were from emergency department and 19 (0.73%) of the requested samples source were not specified. From these requests 842 (32.3%) patients were males and 1497 (57.5%) patients were females and sex was not specified on 267 (10.3%) patients. Overall 742 (28.5%) hematology laboratory errors were detected, of which 560 (75.5%) were pre-analytic, 14 (2%) analytical, 168 (22.6%) post-analytical errors. The highest frequency of pre-analytical errors samples were from emergency (23.6%), followed by in patient ward (23.4%), outpatient (20.7%) and from private wing (17.2%). Out of all the required information, the patient’s identity number and the laboratory test being ordered were present in 2606 (100%) of request forms. The patient’s age was not supplied in 298 (11.5%) test request forms, as listed in Table 1

**Table 1:** Characteristics of the laboratory request forms submitted to the hematology laboratory and errors at St. Paul’s Hospital Millennium Medical College, Addis Ababa, Ethiopia

Documented items	Well written items (%)	Number of errors (%)
Patient ID number	2606 (100)	0 (0)
Patient name	2600 (99.8)	6 (0.2)
Ward/clinic name	2587 (99.3)	19 (0.7)
Request type	2515 (96.5)	91 (3.5)
Date of request	2440 (93.6)	166 (6.4)
Gender	2340 (89.7)	266 (10.3)
Age of patient	2308 (88.5)	298 (11.5)
Physician name	391 (15)	2215 (85)
Physician signature	1764 (67.7)	842 (32.3)
Clinical detail	779 (29.9)	1827 (70.1)

N = 560

#### Pre-analytical, analytical and post-analytical errors

We observed all hematology tests such as complete blood count (CBC), erythrocyte sedimentation rate (ESR) and coagulation tests like activated partial thrombo plastine time (APTT), prothrombin time (PT) and partial thrombin time (PTT). Of the total hematology specimen ordered during the study period, 1509 (58%) were from OPD, 529 (20.3%) from in patient department, 386 (15%) from emergency department, 163 (6.3%) from the private wing, and 19 (0.73%) did not specify the location. Overall, 742 (28.5%) laboratory errors were detected. Of this error of figures, pre-analytic had made contributions of 560 (75.5%), analytical 14 (2%), and post-analytical 168 (22.6%). The highest frequency of pre-analytical problems requests were from emergency (23.6%) whereas the frequencies the private wing (17.2%). The most frequently occurring pre-analytical problems were inadequate sample collection, with a frequency of 274 (48.9%) followed by hemolysis 193 (34.5%) as it is shown in Table 2.

**Table 2:** Types of pre-analytical hematology laboratory errors from different departments in SPHMMC, Addis Ababa, Ethiopia

Categories	Requests N (%)	Emergency N (%)	Inpatient N (%)	Outpatient N (%)	Private wing N (%)	No location N (%)
Total	2606	386 (15)	529 (20.3)	1509 (58)	163 (6.3)	19 (0.73)
Pre-analytical	560 (21.5)	91 (23.6)	123 (23.3)	311 (20.7)	28 (17.2)	7 (35)
Hemolysis	193 (34.5)	10 (11)	59 (48)	122 (39.2)	1 (3.6)	1 (14.3)
Clotted	13 (2.3)	5 (5.5)	5 (4.1)	1 (0.3)	2 (7.1)	0 (0.0)
Mislabeled	32 (5.7)	28 (30.8)	1 (0.8)	1 (0.32)	2 (7.1)	0 (0.0)
Over sample	45 (8.0)	8 (8.8)	20 (16.3)	15 (4.8)	2 (7.1)	0 (0.0)
Inadequate sample	274 (48.9)	30 (32.9)	51 (41.4)	171 (56.3)	19 (67.9)	3 (42.9)
Delayed sample	3 (0.5)	2 (2.2)	1 (0.8)	0 (0.0)	0 (0.0)	0 (0.0)

In the analytical phase, the most frequently detected analytical problem was due to wrong temperature storage of the reagent (42.8%) followed by equipment failure of electric interruption. From post-analytical errors, 126 (75%) were communication errors as shown in Table 3.

**Table 3:** The frequency of analytical and post-analytical errors (N = 182) in hematology laboratory at SPHMMC, Addis Ababa, Ethiopia

Analytical errors	N (%)	Post-analytical errors	N (%)
Wrong reagent storage tem	6 (42.8)	Communication errors	126 (75)
Electric interruption	5 (35.7)	Delayed results	7 (4.1)
Expired reagents	2 (14.2)	Transcription errors	25 (14.8)
Non conformity with QC	1 (7.1)	Data entry errors	9 (5.4)
Total	14 (100)	Lost results	1 (0.6)
		Total	168 (100)

### Discussion

Our study showed that among a total of 2606 hematology samples the total errors were 742 of which 75.5% were pre-analytical, 2% were analytical, and 22% were post-analytical errors. Pre-analytical errors were more common, perhaps caused by rotational duties and workload variety [19, 20]. This study was supported by a similar study conducted in Padua Laboratory, which described pre-analytical errors of 68.2%, analytical of 13.3%, and post of 18.5%, however, the difference of results in analytical errors, may be due to a shortage of internal quality control (QC) systems in our study [10, 21, 22], or because of systemic errors due to inherent technical problems, since accuracy and precision tests were also not performed.

As compared to the study done in Italy [23], clotting in our study is very low, 13 (2.3%). This may be because of the use of ready-made test tubes coated with anticoagulant. The higher result of hemolysis and insufficient volume of blood in the current study may be due to samples collected by non-laboratory professionals who did not recognize collecting samples by correct techniques.

The proportions of pre-analytical errors were the highest in the emergency services (23.5%), followed by inpatient and outpatient service, with 23.2 and 20.6% respectively. The result was higher than other study [2] 3.32% (n = 92), and 1.55% (n = 43), respectively, and may be a result of our professionals being too busy to collect the specimens properly.

The evaluation of hematology request forms showed a well-documented patient name and this result is supported the study done in Nigeria. These forms rated low in clinical diagnosis, recorded just as compared to the study done in Nigeria [6, 24]. The present study also showed that age and gender were left off of the forms at rate of 14.4 and 10.3%, respectively. The finding is low as compared to the study done in Ghana, which 25.6% of patient age and 67.3% of gender were missed [25].

Clinical detail was provided on only 29.8% of the request forms, which varied greatly from the results of the study conducted in Ghana, showing 77.3%. This result may indicate that our clinicians do not have a habit of writing clinical details.

The present study showed that the name of the physician requesting the tests was provided on 391 (15%) forms, and 1764 (67.7%) forms were signed. In a similar study, 55.4% of the forms provided the name of physician and 75.7% were signed. However, higher result of written date of requisition was observed in the current study 2440 (93.6%) relative to the previous, 62.7% [25]. This low result of physician identification in our case may be due to requests ordered by staffs other than a physician. We therefore demonstrated that laboratory requisition forms were not adequately completed by clinicians. Based on our findings post-analytical errors were 22.5%. From all post-analytical errors, communication errors were the highest at 126 (75%). This indicated that the laboratory did not have a strongly linked system with clinicians in the present study.

Since the pre- and post-analytical errors contribution are very high, this suggests better co-operation with clinicians is needed. In addition, provision of training on sample collection and transportation to clinicians and technicians/technologists is advisable.

## Limitation of the study

Our study has some limitation like lack of similar studies in Ethiopia which made difficult for getting more information on the sample size calculation specially and also possible comparison nationwide. In the case of some variables; like hemolysis and icterus samples, measurements were made by visual observation which may lead to inter-personal bias.

## Abbreviations

AIDS: Acquired Immuno Deficiency Syndrome
AOR: adjusted odd ratio
APTT: activated partial thrombo plastine time
CBC: complete blood count
CI: confidence interval
COR: crude odd ratio
ESR: erythrocyte sedimentation rate
OPD: out patient department
PT: prothrombin time
PTT: partial thrombin time
SPHMMC: St. Paul’s Hospital Millennium Medical College
SPSS: Statistical Package for Social Science
